# Serum Health Biomarkers in African and Asian Elephants: Value Ranges and Clinical Values Indicative of the Immune Response

**DOI:** 10.3390/ani10101756

**Published:** 2020-09-27

**Authors:** Katie L. Edwards, Michele A. Miller, Jessica Siegal-Willott, Janine L. Brown

**Affiliations:** 1 Center for Species Survival, Smithsonian Conservation Biology Institute, 1500 Remount Road, Front Royal, VA 22630, USA; BrownJan@si.edu; 2 Department of Science and Innovation-National Research Foundation Centre of Excellence for Biomedical Tuberculosis Research, South African Medical Research Council Centre for Tuberculosis Research, Division of Molecular Biology and Human Genetics, Faculty of Medicine and Health Sciences, Stellenbosch University, Cape Town 8000, South Africa; miller@sun.ac.za; 3 Department of Wildlife Health Sciences, Smithsonian Institution’s National Zoological Park and Conservation Biology Institute, Washington, DC 20008, USA; siegalwillottj@si.edu

**Keywords:** Acute phase proteins, acquired immunity, cytokines, Elephas maximus, ELISA, equine, innate immunity, Loxodonta africana, reference intervals, serum chemistries

## Abstract

**Simple Summary:**

Biomarkers are biological molecules found in the blood or other fluids or tissues that can indicate normal or abnormal processes or disease. Developing tools to measure biomarkers that indicate immune function and establishing concentrations observed within a species is an important first step in their use for managing health and understanding disease processes. Here we report assays, observed value ranges, and concentrations during illness or injury for seven immune biomarkers measured in the serum of African and Asian elephants under human care. Concentrations were variable in both clinical and non-clinical samples, but all seven biomarkers were elevated in at least one case and most increased in response to routine vaccination in a single Asian elephant. These tools provide an exciting avenue for monitoring health status and helping diagnose and treat health problems in wildlife species, like elephants.

**Abstract:**

Serum biomarkers indicative of inflammation and disease can provide useful information regarding host immune processes, responses to treatment and prognosis. The aims of this study were to assess the use of commercially available anti-equine reagents for the quantification of cytokines (tumor necrosis factor-alpha (TNF-α), interferon-gamma (IFN-γ), interleukins (IL) 2, 6, and 10) in African (*Loxodonta africana*, n = 125) and Asian (*Elephas maximus*, n = 104) elephants, and alongside previously validated anti-human reagents for acute-phase proteins (serum amyloid A and haptoglobin), calculate species-specific biomarker value ranges. In addition, we used opportunistically collected samples to investigate the concentrations of each biomarker during identified clinical cases of illness or injury, as a first step to understanding what biomarkers may be useful to managing elephant health. Immune biomarkers were each elevated above the calculated species-specific value ranges in at least one clinical case, but due to variability in both clinical and non-clinical samples, only serum amyloid A was significantly higher in clinical compared to non-clinical paired samples, with tendencies for higher TNF-α and IL-10. We also detected increased secretion of serum amyloid A and all five cytokines following routine vaccination of a single Asian elephant, indicating that these biomarkers can be beneficial for studying normal immune processes as well as pathology. This study indicates that assays developed with commercial reagents can be used to quantify health biomarkers in wildlife species and identifies several that warrant further investigation to elucidate immune responses to various pathologies.

## 1. Introduction

Serum biomarkers indicative of immune function are important health assessment tools in human medicine [1,2,3,4,5] and studies of laboratory and domesticated animal species [6,7,8,9,10,11]. Measurements of acute-phase proteins (APPs) and cytokines aid in detecting pathologies, understanding disease processes and susceptibility, monitoring disease progression, and assessing the efficacy of treatments. APPs form an integral part of the acute-phase response, contributing to the innate immune system. Changes in APPs have been observed in cases of inflammation, infection, neoplasia, stress and trauma, playing a role in promoting healing, and restoring homeostasis [12]. Prior to activation, APPs are generally present in negligible amounts and characterized by the speed and scale of production [7]. With some, a rapid, high magnitude response is associated with acute inflammatory events. Others of moderate concentration remain elevated for more prolonged periods, associated with chronic inflammation. Thus, the evaluation of APPs in serum enables interpretation of the clinical progression of inflammatory responses, and the potential to distinguish between chronic and acute conditions [13]. Furthermore, the relatively rapid response of the acute-phase response makes measuring APP concentrations useful for identifying sub-clinical disease before clinical signs are manifested [12]. APPs have been measured in a variety of wildlife species in recent years, including Asian [14,15,16] and a single African elephant [17] to assess the response to pathologies such as elephant endotheliotropic herpesvirus (EEHV), pododermatitis, trauma, and infection. In elephants, serum amyloid A (SAA) is considered to be a major APP, increasing rapidly in response to acute inflammation, whereas haptoglobin (HP) responds more moderately and may be reflective of more chronic inflammation [15].

Cytokines are protein mediators of the immune response, associated with recruiting, proliferating, activating, differentiating, and otherwise regulating immune cells. Cytokines may be pro-inflammatory, secreted at the beginning of an immune response, or anti-inflammatory, secreted to downregulate the immune response and prevent over-activation. There are numerous cytokines associated with cell-mediated, humoral, and innate immune responses, which can be highly informative regarding immune activation and progression in response to a variety of pathologies. Typically, Th1 cytokines such as IFN-ɣ, IL-2, and TNF-α stimulate cell-mediated immunity to help combat intracellular pathogens (e.g., viruses), whereas Th2 cytokines, including IL-10 and IL-6, promote humoral immune responses, targeting extracellular pathogens (e.g., extracellular bacteria and parasites). Indeed, the combination of cytokines produced can reflect the type and stage of the immune response [18], as well as provide prognostic information regarding the likelihood or progression of disease [19,20]. Some cytokine mRNAs have been characterized in Asian elephants [21,22] and studied previously [23,24,25,26,27]; however, measures of circulating protein concentrations through different pathological processes are lacking, as are typical values observed within a species or population to better interpret results.

Several health problems affect elephants both in situ and ex situ that could benefit from a better understanding of underlying disease processes and improved tools for detection and monitoring. Elephant endotheliotropic herpesvirus hemorrhagic disease (EEHV HD) affects almost one in four Asian elephant calves born in zoos globally [28], as well as African elephants [17,29,30], and in captive and wild populations in Asia [31,32,33,34,35,36,37]. One proposed hypothesis for the severity of EEHV HD could be that immunologically naïve calves fail to mount an effective immune response to keep up with viral replication [38]. A better understanding of how the host immune system responds to the virus would therefore be beneficial and could help assess the efficacy of treatment and novel vaccination options, once they become available. Elephants are also susceptible to infection with *Mycobacterium tuberculosis*, the same causative agent as that for human tuberculosis (TB). Around 10% of elephants currently residing in North America have tested positive for TB, with cases also identified in Europe [39,40], Australasia [41], Africa [42,43], and Asia [44,45,46,47,48,49,50,51]. Although active disease can be detected via culturing trunk secretions, it is not possible to diagnose subclinical infection with current methodologies. Understanding the host immune response to infection could yield important information regarding the transition from subclinical to active infection, susceptibility to disease, and the efficacy of various treatments.

In addition to infectious disease, biomarkers indicative of inflammation and immune function would improve the ability to detect and manage other common pathologies. A recent survey of elephant health in North America highlighted gastrointestinal issues, skin lesions and wounds, lameness and foot lesions, eye issues, tusk and sulcus injuries, and dental disorders as prevalent pathology types [52]. Similarly, Miller et al. [53] highlighted injuries, parasitism, gastrointestinal disease, and infectious disease as being responsible for elephant morbidity and mortality in Asia. All these pathologies are associated with inflammation and immune activation, and therefore tools to help with sub-clinical detection, diagnosis and treatment would benefit overall elephant health. To-date, published reference ranges only exist for two APPs, SAA and HP, in Asian elephants [16]; none have yet been established for African elephants. The goal of this study was to establish and validate assays for several other candidate biomarkers using commercially available reagents and calculate reference intervals for African and Asian elephants in North American zoos. Although reference interval calculations were conducted using standard methodology [54], we hereafter refer to these as species-specific value ranges because underlying health issues without overt clinical signs cannot be ruled out. Specifically, we analyzed APPs (SAA and HP) and cytokines (tumor necrosis factor-alpha (TNF-α), interferon-gamma (IFN-ɣ), interleukins 2 (IL-2), 6 (IL-6) and 10 (IL-10)) and compared values to individuals exhibiting clinical signs of illness or injury, or prior to death. To add to the current literature available, we also report serum chemistry value ranges from the same population, representing a large sample-set analyzed in the same laboratory.

## 2. Materials and Methods

### 2.1. Subjects, Sample Collection, and Assessment of Health Status

Single serum samples were obtained from 229 elephants housed at 69 institutions in North America. Subjects included 125 African (18 male, 107 female) and 104 Asian (18 male, 86 female) elephants aged 4 to 65 years. Additional serum samples were collected opportunistically from elephants with active clinical pathology at the time of sample collection (n = 10) and prior to death (n = 10), and from the same individuals when no clinical signs were present, a minimum of one month before or after the clinical sample. Weekly serum samples collected prior to and following routine vaccination with tetanus toxoid (1 mL intramuscularly) and Imrab 3 (1 mL intramuscularly) were also collected from a female Asian elephant, aged 21 years. Active clinical cases and conditions present at necropsy were determined by the attending veterinarian or pathologist. Blood samples were collected according to phlebotomy protocols at each institution, typically from an ear vein while the elephant was under behavioral restraint. After being allowed to clot at room temperature for 1 h, serum was separated and frozen at −20 °C before shipment to the Smithsonian Conservation Biology Institute for analysis. This research was approved by the Smithsonian National Zoo (NZP-ACUC #11-10, #15-03, and #18-18) and where applicable, was reviewed and approved by participating zoo research and animal care committees.

### 2.2. Acute Phase Protein Analysis

SAA and HP were measured using an RX Daytona automated clinical chemistry analyzer (Randox Industries-US Ltd., Kearneysville, WV, USA). Commercially available reagents, calibrators, and two-level controls were used (Eiken Chemical Co. Ltd., Tokyo, Japan and Tridelta Tri-DD, Boonton, NJ, USA, respectively). The technical ranges were 0.1 to 500 mg/L and 0.01 to 2.5 mg/mL, respectively. The analyzer was subject to routine quality control measurements throughout the study, with normal and elevated controls for each analyte maintained within 2 standard deviations (SD) of the respective lot-specific target value. Samples were typically analyzed neat, but some with HP above the technical range were diluted 1:5 or 1:10 in calibrator diluent as needed.

### 2.3. Cytokine Enzyme Immunoassays

TNF-α was measured using an equine TNF-α enzyme immunoassay (EIA) (Invitrogen ESS0017; Thermo Fisher Scientific, Frederick, MD, USA) according to the manufacturer’s instructions (Table 1). In brief, anti-equine TNF-α coating antibody was diluted in carbonate-bicarbonate buffer (0.2 M, pH 9.4), and 100 µL added to each well of a 96-well microtiter plate (Costar, Corning Life Sciences, Tewkesbury, MA, USA). Following incubation at room temperature overnight, coating antibody solution was aspirated, and wells were blocked with a Dulbecco’s phosphate-buffered saline (D-PBS; 8 mM Na_2_HPO_4_, 2 mM KH_2_PO_4_, 0.14 M NaCl, 10 mM KCl, pH 7.4.) solution containing 4% bovine serum albumin (BSA) and 5% sucrose, for a minimum of 1 h. Blocking buffer was aspirated and 50 µL standards, controls, or samples added in duplicate, before incubation for 1 h at room temperature while shaking at 500 RPM. Recombinant equine TNF-α standards were serially diluted in reagent diluent (4% BSA in D-PBS, pH 7.4), and additionally diluted to provide high and low concentration control samples. Serum was typically run neat or diluted up to 1:20 in reagent diluent as needed. Plates were then washed three times (0.05% Tween^TM^-20 in D-PBS, pH 7.4), before 100 µL per well of anti-equine TNF-α detection antibody was added and incubated for a further 1 h at room temperature while shaking at 500 RPM. Following a further three washes, 100 µL per well streptavidin-horseradish peroxidase (diluted 1:400 in reagent diluent) was added and incubated for 30 min at room temperature while shaking at 500 RPM. After a final wash step, 100 µL of chromogenic substrate solution was added per well, incubated in the dark for 20 min at room temperature, stopped with 100 µL stop solution and absorbance measured at 450 nm with a reference of 570 nm.

IFN-ɣ, IL-2, and IL-10 were measured using equine Duosets (R&D Systems, Inc., Minneapolis, MN, USA) according to modified manufacturer’s instructions (Table 1). In brief, goat anti-equine coating antibody was diluted in phosphate-buffered saline (PBS; 137 mM NaCl, 2.7 mM KCl, 8.1 mM Na_2_HPO_4_, 1.5 mM KH_2_PO_4_, pH 7.4), and 100 µL was added to each well of a 96-well microtiter plate (Costar). Following incubation at room temperature overnight, the coating antibody solution was aspirated, and plates were washed three times with wash buffer (0.05% Tween ^TM^-20 in PBS, pH 7.4) and then blocked with a 4% BSA, 5% sucrose PBS solution for a minimum of 1 h. Blocking buffer was aspirated and 50 µL standards, controls, and samples added in duplicate, before incubation for 2 h at room temperature while shaking at 500 RPM. Recombinant equine standards and control samples were diluted in 50% fetal bovine serum (FBS) in reagent diluent (1% BSA in PBS, pH 7.4). Serum samples were typically run neat or diluted up to 1:20. Plates were then washed three times before 100 µL per well of biotinylated goat anti-equine detection antibody (diluted in reagent diluent without FBS) was added and incubated for a further 2 h at room temperature while shaking at 500 RPM. Following a further three washes, 100 µL per well streptavidin-horseradish peroxidase (diluted 1:200 in reagent diluent without FBS) was added and incubated in the dark for 20 min at room temperature. After a final wash step, 100 µL of substrate solution (high kinetic TMB peroxidase substrate, Moss, Inc., Pasadena, MD, USA) was added per well, incubated in the dark at room temperature, stopped with 50 µL of stop solution (1N HCl), and absorbance measured at 450 nm with a reference of 570 nm. Anti-equine IL-1β and IL-4 Duoset antibodies (Table 1) also showed good cross-reactivity with both African and Asian elephant serum following a similar protocol except for dilution of detection antibody in 2% FBS in reagent diluent. However, intermittent issues with elevated background prevented the measurement of IL-1β and IL-4 for the remainder of this study. IL-6 was measured using goat anti-equine antibodies (Table 1) with a protocol similar to that described for Duosets, except that standards and controls were diluted in reagent diluent without FBS. All EIAs were biochemically validated according to the manufacturer’s recommendations prior to the start of the study by performing spike and recovery and linearity assessments with elephant serum [55,56]. Inter-assay coefficients of variation were maintained below 15% for high and low concentration controls on all assays.

### 2.4. Serum Chemistries

Twenty-two serum analytes (Alanine aminotransferase, albumin, alkaline phosphatase, aspartate aminotransferase, bilirubin, calcium, carbon dioxide, chloride, cholesterol, creatine kinase, creatinine, gamma glutamyl transferase, glucose, iron, lactate dehydrogenase, magnesium, phosphorus, potassium, sodium, total protein, triglycerides, and urea nitrogen) were measured in each sample using a Dimension^®^ Xpand Plus automated clinical chemistry analyzer (Siemens Medical Solutions USA, Inc., Malvern, PA, USA). The analyzer was subject to routine quality control measurements throughout the study, with two-level controls maintained within manufacturer specifications.

### 2.5. Value Range Calculation

Value ranges for African and Asian elephants under human care were calculated for each serum biomarker according to reference interval guidelines from the American Society for Veterinary Clinical Pathology [54]. Value ranges for serum chemistry parameters were generated using the robust method, and immune biomarkers using the nonparametric method, all using the “referenceIntervals” package [57] in R statistical software [58], version 3.6.1. Outlying values were identified using Cook’s distance and were removed prior to calculation; all value ranges represent 95% of the population and are reported with 90% confidence intervals.

### 2.6. Statistical Analyses

Each of the 29 analytes (2 APP, 5 cytokine, and 22 serum chemistry) used for determining value ranges were compared by species (125 African and 104 Asian) using a Mann–Whitney Wilcoxon test. A Wilcoxon signed-rank test was used to compare each of the seven immune biomarkers (2 APP, 5 cytokine) in individuals that exhibited clinical signs of illness to control samples taken from the same individual when no clinical signs were exhibited. All analyses were conducted in R [58], version 3.6.1, with alpha set to 0.05.

## 3. Results

**Figure 1:**
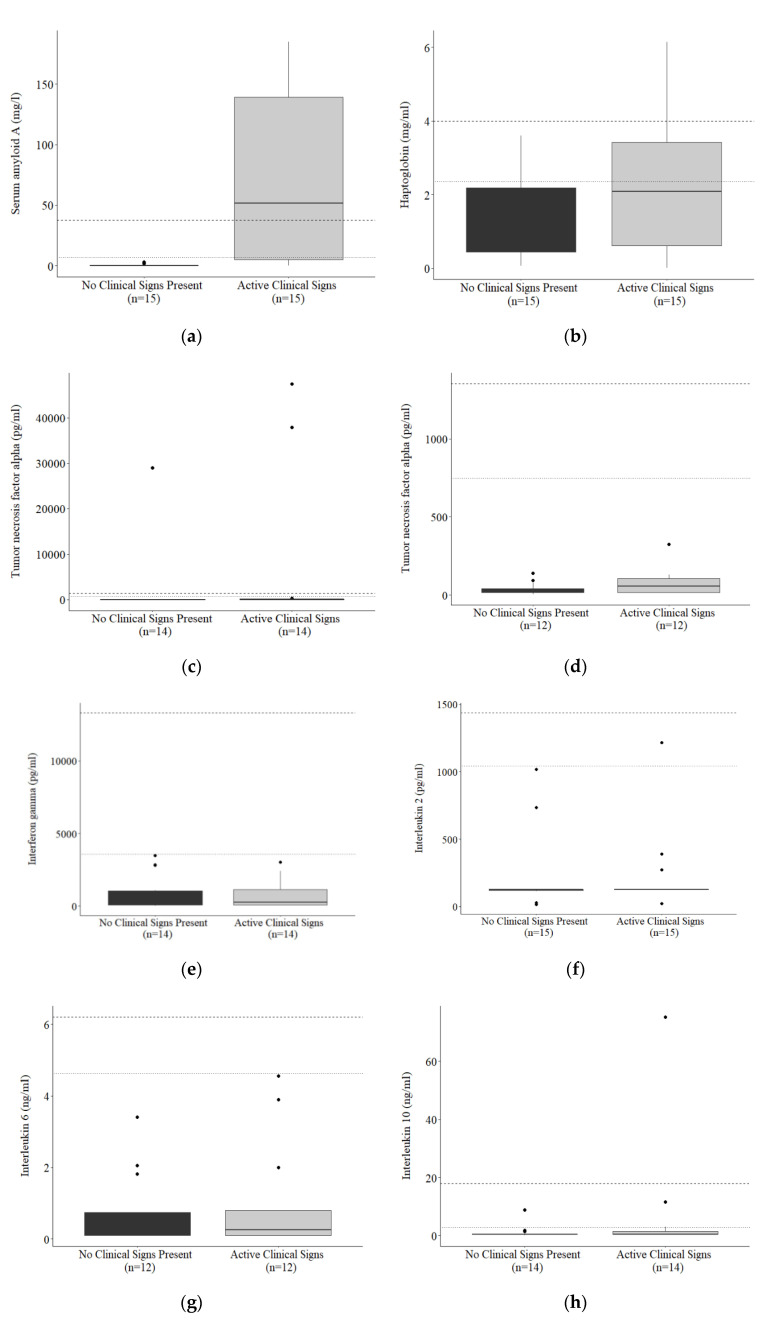
Paired concentrations of serum biomarkers (**a**) SAA (*p* = 0.004), (**b**) HP, (**c**) TNF-α (*p* = 0.021), (**d**) TNF-α outliers removed, (**e**) IFN-ɣ, (**f**) IL-2, (**g**) IL-6, and (**h**) IL-10 in individuals with or without active clinical signs present. The calculated upper limits of the species-specific value ranges are denoted by dashed (*E.m.*) or dotted (*L.a.*) horizontal lines.

Concentrations of SAA (*p* < 0.001), HP (*p* < 0.001), TNF-α (*p* = 0.011), IFN-ɣ (*p* = 0.025), and IL-2 (*p* = 0.029) were higher in Asian elephants, with IL-6 (*p* = 0.464) and IL-10 (*p* = 0.139) not differing between species (Table 2). Value ranges calculated for APPs and cytokines are presented in Table 2. A summary of APP and cytokine concentrations in individuals with active clinical signs of injury or illness is shown in Table 3, and around the time of death in Table 4. SAA was elevated above species-specific value ranges in 10/12 clinical cases (Table 3) and 5/10 individuals at or leading up to death (Table 4), with the highest concentrations (251.82 mg/L) observed in an African elephant with bronchopneumonia. Overall, SAA was higher in individuals with active clinical signs of illness or injury compared to the same individuals when no clinical signs were apparent (*p* = 0.004; Figure 1a). HP was elevated in 5/12 clinical cases (Table 3) and 2/10 individuals leading up to death (Table 4), with the highest concentrations observed in an Asian elephant that had become recumbent and unable to rise. Average HP concentrations did not differ between individuals with clinical signs compared to those without (Figure 1b) (*p* = 0.477).

**Figure 2:**
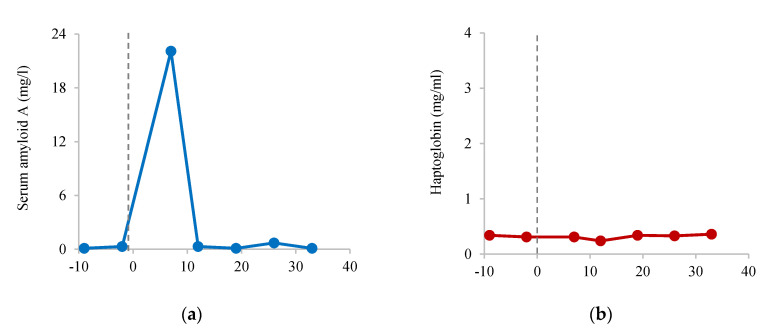

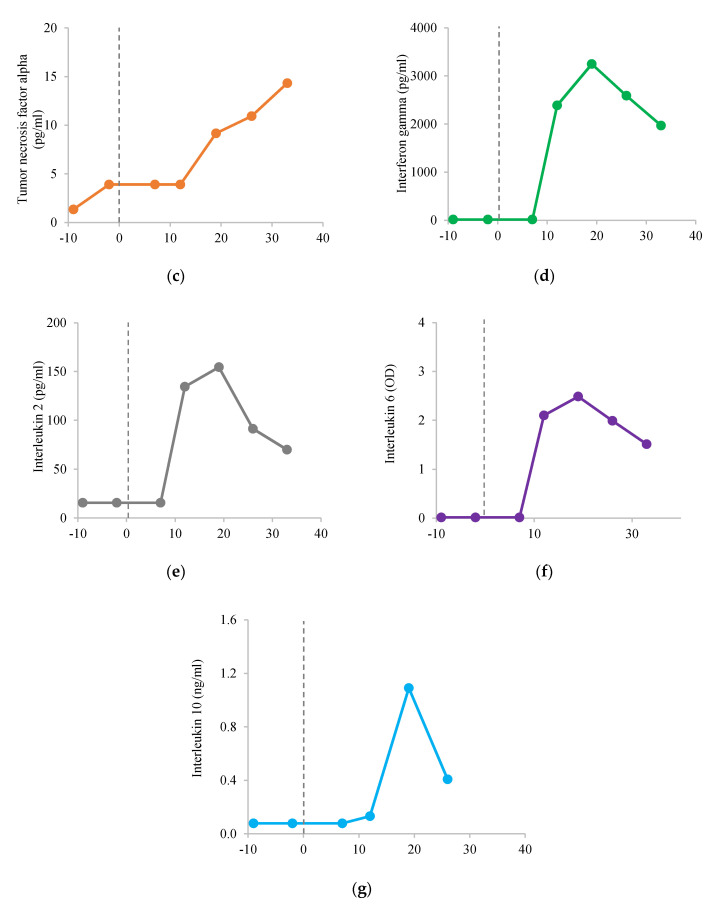
Response to tetanus and rabies vaccination in an adult female Asian elephant, aged 21 years: (**a**) SAA, (**b**) HP, (**c**) TNF-α, (**d**) IFN-γ, (**e**) IL-2, (**f**) IL-6, and (**g**) IL-10. Dashed line denotes the day of vaccination, day 0.

Cytokine concentrations were elevated above the species-specific value ranges in several clinical cases (Table 3), including pododermatitis, systemic infection, acute lameness, ventral edema, and tusk infection. Overall, TNF-α was higher in individuals with active clinical signs of illness or injury compared to when no clinical signs were apparent (*p* = 0.021), IL-10 tended to be higher during active clinical cases (*p* = 0.059), whereas IFN-ɣ, IL-2, and IL-6 did not differ significantly despite higher mean concentrations in clinical cases (Figure 1). One female African elephant, representing two clinical cases (tusk injury and infection), had TNF-α concentrations considerably higher than all other individuals tested. When samples from this individual were excluded from the paired comparison, TNF-α in clinical samples only tended to be higher than non-clinical samples (*p* = 0.068). In many cases, cytokines were only mildly elevated and did not exceed the upper value range or were below the detection limit of the assays; in no cases were all seven biomarkers elevated concurrently. By contrast, routine vaccination of a female Asian elephant against tetanus and rabies stimulated an increase in SAA and all cytokines (Figure 2). Concentrations were all below assay detection at nine and two days prior to vaccination but increased from 7 (SAA), 12 (IFN-γ, IL-2), or 19 (TNF-α, IL-10) days post-vaccination. IL-6 showed a similar pattern to that of IFN-γ and IL-2, but peak concentrations could only be extrapolated, so instead are presented as optical density (OD).

Descriptive statistics and calculated value ranges for 22 serum biochemistries are provided in Table 5. Compared to previously published data [59], value ranges in our study were narrower for albumin, alkaline phosphatase, bilirubin, calcium, cholesterol (Asians only), creatinine, gamma glutamyl transferase, glucose (Africans only), iron, magnesium, potassium, total protein, and urea nitrogen. Except for alkaline phosphatase, aspartate aminotransferase, creatinine, carbon dioxide, and glucose, all other biochemistries differed significantly between species (*p* ≤ 0.04).

## 4. Discussion

The determination of circulating biomarker concentrations is useful for identifying abnormal processes or disease and is increasingly used in human and animal medicine. However, a lack of validated assays and information on typical concentrations observed has hampered the use of these biomarkers in wildlife medicine. Here, we report the development of enzyme immunoassays for analysis of five cytokines in African and Asian elephants using commercially available anti-equine antibodies. We also determined species-specific value ranges for these cytokines and two APPs in African and Asian elephants in human care and quantified each of these biomarkers in a variety of active clinical cases to investigate their value in identifying underlying immunological changes. Elevated concentrations above species-specific value ranges were observed for all biomarkers in association with at least one active clinical case, but not all. With all clinical cases combined, SAA was significantly elevated over paired non-clinical cases, with tendencies for higher TNF-α and IL-10.

This study focused on the ability to measure cytokines at the protein level (i.e., secreted) as opposed to cytokine expression at the RNA level, which has been used to investigate tuberculosis [21,23,24,26] and EEHV [27] infection in Asian elephants previously. The main advantage of this approach is that we can utilize cryopreserved serum samples that are already collected for routine veterinary and management purposes, allowing a longitudinal perspective on changes in immune function required for a better understanding of physiological and pathological processes. Serum is regularly collected from elephants for routine reproductive and health monitoring, and quantification of circulating biomarkers would be feasible without the need for additional processing to stabilize RNA to prevent degradation or changes in gene expression ex vivo. Enzyme immunoassays also have an advantage in that the equipment and reagents are relatively inexpensive, so they have the potential for use around the world.

The value range calculated for SAA in Asian elephants was similar to that previously published by Isaza et al. [16], based on a sample of 35 Asian elephants with the upper reference interval at 47.5 mg/L and upper confidence interval at 55.9 mg/L. Clinically abnormal elephants have been described with levels from 30 to 300 mg/L (C. Cray, personal.commumication). In contrast, the HP value range calculated from this dataset was higher than that of Isaza and colleagues, who reported an upper reference interval of 1.13 mg/mL and upper confidence interval of 1.26 mg/mL. For the Asian elephants in our population, the HP value range was more than three times higher. Although the age ranges of elephants from these two sample sets were similar (this study, 4 to 65 years; Isaza et al., 3 to 66 years), higher HP concentrations could perhaps indicate more chronic conditions may have been present in our larger dataset. It is important to note that although we used established reference interval methodology, our calculated value ranges cannot be considered healthy reference intervals as there may have been underlying conditions in this population that were undetected. We used a statistical technique to remove outliers—a conservative approach recommended where underlying health conditions cannot be readily ascertained [54], so the presented ranges likely are broader than for a truly healthy sample, and instead represent values that may be observed within a population under human care. Due to our sample size and recommendations that reference intervals are not reliable with less than 20 individuals, we were unable to separate data to calculate value ranges separately by sex or by age category, which could be explored further for potential differences once samples from additional individuals are available. Similarly, because our dataset contains only individuals from an ex situ population, further investigation will be required to determine how these may compare to free-ranging elephants.

African elephant reference intervals/value ranges for APPs have not been published previously, but Bronson et al. [17] reported concentrations of SAA and HP in an African elephant with EEHV hemorrhagic disease. In that case, a peak concentration of 88.4 mg/L SAA was observed on Day 2 of illness, and 3.4 mg/mL HP on Day 14, both of which exceeded the value ranges that we have calculated for African elephants in this study. APPs increase as part of the innate inflammatory acute phase response; typically, major APPs increase 10 to 1000-fold within 24 to 48 h of a stimulus, moderate APPs 5 to 10 fold within a few days, and minor APPs increase more slowly, peaking around two-fold [12] after four to six days [15]. Here we describe increases in SAA up to 30-fold, and HP two to three-fold above the upper value range. Similar to other reports [15,16], these data suggest that in elephants, SAA may constitute a major APP associated with acute inflammation, whereas HP may reflect more chronic changes.

Although clear differences in cytokine concentrations were not detected between paired clinical and non-clinical samples, all cytokines were elevated in at least one clinical case, including lameness, pododermatitis, systemic and tusk infections, and tusk injury. Foot and joint pathology is common in elephants, representing 11.7% (foot lesions) and 12.3% (lameness and/or stiffness) of clinical events reported for the same population over the course of 12 months [52], and so improved tools for managing these conditions would be beneficial to elephant well-being. Here we detected increased concentrations of all cytokines associated with lameness and/or pododermatitis; however, no single biomarker was consistently elevated in all cases. Across species, researchers have explored different cytokines in relation to similar pathology. For example, chronically lame dairy cows exhibited higher concentrations of TNF-α and IFN-γ [60], horses with laminitis had increases in both Th1 and Th2 cytokines [61], vervet monkeys (*Chlorocebus aethiops sabaeus*) with osteoarthritic changes secrete increased amounts of inflammatory cytokines [62], and dogs with pododermatitis had significant over-expression of IL-6 and IL-10 [63]. Furthermore, cytokine over-expression can play a role in both rheumatoid [64] and osteoarthritis [65], and cytokine-modulating treatments are utilized in humans [66] and some animal species [67,68]. Additional research targeting specific musculoskeletal pathologies common in elephants, and how different immune biomarkers are up- or down-regulated throughout the progression of disease and response to treatment would be beneficial to inform disease management.

The immune response to infection can also be detected through altered cytokine production. For example, IFN-γ, TNF-α, and IL-10 expression is increased in Asian elephants seropositive for tuberculosis [26], and TNF-α and IFN-γ are upregulated in in vitro stimulated immune cells from elephants with EEHV [27]. In this study, an Asian elephant with a systemic infection exhibited elevated IL-10, IFN-γ, and TNF-α compared to paired non-clinical sample concentrations in the same individual. This is similar to models of systemic inflammation where increased TNF-α, IFN-γ, IL-6, and IL-10 have all been reported [69]. Localized infections also elicit immune responses that can be measured in circulation. Anatolian buffaloes with infectious skin lesions exhibit elevated TNF-α and IL-6 compared to healthy controls [70], and here tusk infections were associated with increased pro- and anti-inflammatory cytokines TNF-α and IL-10, respectively.

Differences in cytokine concentrations between clinical and non-clinical samples were not always clear in this study. However, elevated biomarker concentrations in non-clinical samples could reflect sub-clinical levels of immune activation [71,72] or age-related degenerative changes in immune function [73,74]. Detection of cytokines in clinically normal animals has also been reported when investigating specific pathologies in other species. For example, IL-6 and IL-8 were significantly higher in dogs with acute diarrhea but were also detectable in 28% and 44% of non-diarrheic dogs, respectively [75]. Our results also indicate individual differences/outlier values that are yet to be explained. In one case, a female African elephant with a tusk injury and subsequent chronic infection exhibited substantially elevated TNF-α compared to all other individuals, but on retrospective testing, concentrations were elevated prior to the injury and persisted within a range of 17,000 to 66,000 pg/mL for at least five years before and after this event. TNF-α is a pro-inflammatory cytokine associated with trauma and infection but has also been associated with autoimmune processes [76]. Chronically elevated concentrations such as these may indicate underlying pathology that warrants further investigation. Clearly, additional studies are required to improve our understanding of immune biomarkers in elephants, ideally with larger sample sizes specifically selected for particular types of pathology and incorporating longitudinal sample collection to distinguish individual variability from clinical changes.

In addition to assessing changes relating to pathology, measuring serum biomarkers indicative of the immune response can also be beneficial to understanding and monitoring the response to vaccination [77], to determine whether the immune system is responding appropriately. Here, we report biomarker responses to tetanus and rabies vaccination of a female Asian elephant that may indicate the progression and development of acquired immunity. Although concentrations of SAA increased by seven days post-vaccination, the cytokine response was not detected until at least 12 days following vaccination, with anti-inflammatory IL-10 being the last to increase. This is a similar timeline to that reported by Humphreys et al. [78], where serum anti-IgG titers to tetanus toxoid increased two to three weeks following vaccination.

Biomarker analyses in humans, livestock, and laboratory species often utilize species-specific antibodies; however, these are generally lacking for most wildlife species. Molecular characterizations of Asian elephant IFN-γ [21], and more recently IL-1β, and IL-8 [22] have paved the way for elephant-specific antibodies [79,80]. However, lack of homologous assays should not preclude exploration of immune biomarkers for understanding health and disease in wildlife. Numerous studies have used commercial reagents in several wildlife species to validate and/or assess population-level differences in cytokines, including the cheetah [81], bottlenose dolphin [82], and pinnipeds [83], setting the basis for more detailed pathology-specific investigations, as has begun for APPs [15,84,85,86,87]. However, reagents must be tested and assays validated before use because not all are suitable (e.g., porcine reagents for Florida manatee [88] and African and Asian elephant (Edwards, unpublished)). The commercially available anti-equine (cytokines) and anti-human (APPs) reagents used in this study successfully cross-reacted with elephant proteins, suggesting they can be used to assess the elephant immune response to inflammation and disease.

Finally, although more widely reported, many of the serum chemistry value ranges calculated in the current study were narrower than those previously published [59]. The reference ranges provided by Fowler and Mikota include data from captive and wild elephants of both species, both sexes and a variety of ages, and are a compilation of at least a dozen sources. Thus, the removal of lab to lab variability in our dataset may have contributed to the narrower value ranges for many analytes. One exception was alanine aminotransferase, where our calculated value range of 0.00 to 8.03 IU/L in African and 0.00 to 5.89 IU/L in Asian elephants had a wider range and a greater maximum than that (1.5 to 3.0 IU/L) reported in Fowler and Mikota [59]. Although the clinical significance of this difference is not clear, higher concentrations can be associated with muscle damage. Of the 22 serum biochemistries included here, 17 differed significantly between African and Asian elephants, highlighting the need to build robust reference intervals independently for these species.

## 5. Conclusions

The ability to measure circulating biomarkers may provide additional information to detect sub-clinical signs of inflammation and provide an insight into the nature of the immune response, which may not be revealed during routine examination or assessment of serum biochemistries and complete blood counts. Here we report validation, value ranges, and clinical values for two APPs and five cytokines, in African and Asian elephants in human care. Although these biomarkers are non-specific, if an animal exhibits an increase in APPs or cytokines, this information could help veterinarians decide if further diagnostic investigations are warranted. By applying multiple different biomarkers including pro-inflammatory and anti-inflammatory, Th1 and Th2 cytokines, and major and minor APPs, the combination could provide valuable information regarding the underlying immune response that can guide further diagnostics, treatment, and prognosis. Additionally, these biomarkers can indicate the immune response to routine vaccination, and so could be used for assessing the response to interventions. Further research is vital to our understanding of immune processes underlying different pathologies, and how biomarker concentrations may differ between age categories, sexes and other physiological states, and between captive and free-ranging animals. These and other potential biomarkers provide an exciting avenue for monitoring of health status and helping diagnose and treat health problems in wildlife species, like elephants, managed under human care.

## Figures and Tables

**Table 1:** Information for cytokine enzyme immunoassays validated for African and Asian elephants.

Cytokine	Supplier	Reagents	Standard Range	Antibody Concentration	
				Capture	Detection
Tumor necrosis factor-alpha (TNF-α)	Thermo Scientific/Invitrogen	ESS0017	3.9–1000 pg/mL *	1:100	1:100
Interferon-gamma (IFN-ɣ)	R&D Systems	Duoset DY1586	15.6–4000 pg/mL *	0.4 μg/mL	0.4 μg/mL
Interleukin 1β (IL-1β)	R&D Systems	Duoset DY3340	125–8000 pg/mL	0.8 μg/mL	0.15 μg/mL
Interleukin 2 (IL-2)	R&D Systems	Duoset DY1613	15.6–4000 pg/mL *	2.0 μg/mL	0.2 μg/mL
Interleukin 4 (IL-4)	R&D Systems	Duoset DY1809	31.2–2000 pg/mL	0.8 μg/mL	0.8 μg/mL
Interleukin 6 (IL-6)	R&D Systems	AF1886/BAF1886/ 1886-EL	0.1–25 ng/mL *	0.4 μg/mL	0.4 μg/mL
Interleukin 10 (IL-10)	R&D Systems	Duoset DY1605	0.078–20 ng/mL *	0.4 μg/mL	0.1 μg/mL

* Assay sensitivity was increased throughout the course of assay development, so samples at the lower end of detection may be higher than the final range given here.

**Table 2:** Descriptive statistics and calculated value ranges (with 90% confidence intervals, CI) for serum acute-phase proteins and cytokines from 125 African (*L.a.*) and 104 Asian (*E.m.*) elephants under human care.

Analyte	Species	Mean	SD	Median	Minimum	Maximum	N ^a^	Value Range	Lower CI ^b^	Upper CI
SAA (mg/L)	*L.a.*	4.16	26.25	0.10	0.10	251.82	123	0.10–6.91	-	6.38–8.38
	* E.m.	16.04	47.05	1.84	0.10	231.92	98	0.10–37.62	-	23.98–53.25
HP (mg/mL)	*L.a.*	1.36	0.74	1.40	0.19	5.45	124	0.21–2.35	0.18–0.23	2.25–2.40
	* E.m.	1.96	1.57	1.83	0.19	10.50	100	0.24–4.00	0.18–0.29	2.95–4.93
TNF-α (pg/mL)	*L.a.*	311.89	1783.69	15.60	15.60	17,381.84	123	15.60–748.10	-	309.31–1031.38
	* E.m.	336.35	1249.48	25.29	15.60	10,484.78	101	15.60–1355.83	-	1319.30–1929.39
IFN-ɣ (pg/mL)	*L.a.*	745.73	2094.26	62.50	62.50	19,176.99	122	62.50–3565.07	-	2424.93–4021.17
	* E.m.	3564.55	16,761.97	62.50	62.50	124,117.26	102	62.50–13,317.40	-	6888.22–22,342.10
IL-2 (pg/mL)	*L.a.*	293.91	866.31	125.00	125.00	8651.83	123	125.00–1043.61	-	762.66–1444.99
	* E.m.	309.88	1303.44	125.00	125.00	12,770.00	96	125.00–1438.85	-	1096.83–2499.38
IL-6 (ng/mL)	*L.a.*	1.85	6.15	0.39	0.39	51.59	116	0.39–4.63	-	4.55–7.04
	*E.m.*	2.02	7.37	0.39	0.39	56.15	93	0.39–6.20	-	2.38–8.92
IL-10 (ng/mL)	*L.a.*	0.92	1.85	0.31	0.31	16.04	120	0.31–2.73	-	1.39–3.53
	*E.m.*	4.25	30.39	0.31	0.31	303.27	99	0.31–18.00	-	17.04–32.16

^a^ Number of samples used for value range calculation after outlier removal. ^b^ For all biomarkers except HP, the lower end of the calculated value range is the limit of detection so no lower CI could be calculated. * Species has higher concentrations at *p* < 0.05 level.

**Table 3:** Acute-phase protein and cytokine concentrations in African (*L.a*) and Asian (*E.m*) elephants with active clinical cases. Numbers in bold exceed the upper end of the calculated value range for that species.

Species	Clinical Event	Age (Years)	Sex	SAA (mg/L)	HP (mg/mL)	TNF-α (pg/mL)	IFN-ɣ (pg/mL)	IL-2 (pg/mL)	IL-6 (ng/mL)	IL-10 (ng/mL)
*E.m*	Pododermatitis	49	F	10.00	2.36	<15.60	117.08	<125.00	<0.39	1.74
*E.m*	Pododermatitis	46	F	**193.80**	**8.00**	**10,484.78**	<15.60	<125.00	<0.39	<0.31
*E.m*	Systemic infection	39	F	**136.00**	0.80	324.84	2419.97	ND	<0.39	**75.27**
*E.m*	Enteritis	3	M	**149.20**	**4.55**	<15.60	ND	<125.00	ND	ND
*E.m*	Lameness	19	F	**137.00**	3.30	<15.60	719.28	<125.00	2.00	<0.31
*E.m*	Lameness	41	F	0.10	0.32	689.34	**124,117.26**	**12,770.00**	**>125.00 ^a^**	**303.27**
*E.m*	Ventral edema	42	F	**51.50**	1.02	55.57	3025.28	1215.14	**>125.00 ^a^**	11.53
*E.m*	Recumbent/unable to rise	36	F	**233.70**	**11.00**	73.04	1129.42	<125.00	<0.39	<0.31
*L.a*	Tusk injury	42	F	**141.40**	**4.65**	**47,475.75**	<62.50	<125.00	<0.39	<0.31
*L.a*	Tusk infection	42	F	**147.40**	**3.55**	**37,935.20**	399.53	<125.00	<0.10	0.38
*L.a*	Tusk infection	34	M	**48.60**	2.09	57.53	1284.34	<125.00	4.55	**3.00**
*L.a*	Septicemia	7	M	**184.90**	**6.15**	121.29	ND	20.85	ND	ND

^a^ Above the measurable range of the current assay system when diluted 1:5, ND: not determined.

**Table 4:** Acute-phase protein and cytokine concentrations in African and Asian elephants prior to death. Numbers in bold exceed the upper end of the calculated value ranges for that species.

Species	Associated Conditions Found at Necropsy	Days Prior to Death	Age (Years)	Sex	SAA (mg/L)	HP (mg/mL)	TNF-α (pg/mL)	IFN-ɣ (pg/mL)	IL-2 (pg/mL)	IL-6 (ng/mL)	IL-10 (ng/mL)
*E.m*	Deteriorating health, anemia, thrombocytopenia, B-cell lymphoblastic leukemia/lymphoma, ventral edema	0	58	F	**42.30**	0.07	128.84	<62.50	<125.00	<3.90	<0.31
*E.m*	Sepsis, dental disease, ulcerative dermatitis	11	37	F	**59.50**	0.01	35.83	1100.66	<125.00	<0.10	<0.31
*E.m*	Deteriorating health, neoplasia: uterine adenocarcinoma, anaplastic sarcoma, lymphoma, uterine adenoma, degenerative osteoarthritis, ventral edema	1	54	F	**207.34**	**6.95**	98.400	<62.50	<125.00	<0.39	<0.31
*L.a*	Degenerative cardiovascular disease, ventral edema/ascites, loss of condition, thoracic abscess	1	43	F	0.10	0.30	<15.60	<62.50	<125.00	<0.10	<0.31
*L.a*	Deteriorating health, degenerative joint disease, recumbent, unable to rise	0	38	F	0.10	1.95	83.82	<62.50	272.42	0.10	0.70
*L.a*	Deteriorating health, degenerative joint disease, recumbent, unable to rise	0	55	F	0.10	2.13	111.02	<62.50	<125.00	<0.10	<0.31
*L.a*	Deteriorating health, degenerative joint disease, recumbent, unable to rise	0	45	F	0.10	0.43	<15.60	<62.50	389.08	<0.10	0.62
*L.a*	Bronchopneumonia, degenerative joint disease	19	32	M	**251.82**	**5.45**	<15.60	429.08	<125.00	<0.39	<0.31
*L.a*	Deteriorating health, pneumonia, pododermatitis, gastrointestinal ulcers, ulcerative dermatitis, ventral edema	7	34	F	**153.94**	0.26	<15.60	<62.50	<125.00	<0.39	<0.31
*L.a*	Deteriorating health, septic peritonitis	16	47	F	5.45	1.49	100.89	<62.50	<125.00	<0.39	<0.31

**Table 5:** Descriptive statistics and calculated value ranges (with 90% confidence intervals, CI) for serum chemistries from 125 African (*L.a.*) and 104 Asian (*E.m.*) elephants under human care.

Analyte	Published Range ^a^	Species	Mean	SD	Median	Min.	Max.	N ^b^	Value Range	Lower CI	Upper CI
Alanine aminotransferase (IU/L)	1.5–3.0	*L.a.	3.62	3.02	4.00	0.00	22.00	114	0.00–8.03	0.00–0.00	7.66–8.67
		*E.m.*	2.51	2.04	2.00	0.00	8.00	92	0.00–5.89	0.00–0.00	4.96–6.49
Albumin (g/dL)	1.5–3.5	*L.a.	2.90	0.50	2.90	1.60	5.50	120	2.29–3.47	2.21–2.39	3.41–3.58
		*E.m.*	2.73	0.33	2.80	1.50	3.50	99	2.23–3.27	2.13–2.30	3.21–3.35
Alkaline phosphatase (IU/L)	60–450	*L.a.*	83.94	39.72	75.00	30.00	231.00	116	22.10–119.83	13.65–29.83	111.87–127.98
		*E.m.*	84.32	43.15	76.50	33.00	322.00	100	29.48–120.84	22.20–37.34	112.69–129.60
Aspartate aminotransferase (IU/L)	15–35	*L.a.*	15.87	12.09	14.00	4.00	124.00	123	0.79–26.04	0.00–2.98	23.71–28.21
		*E.m.*	14.33	7.01	13.00	0.00	39.00	98	2.13–24.34	0.74–3.40	22.69–26.01
Bilirubin (mg/dL)	0.2–1.0	*L.a.*	0.18	0.04	0.18	0.10	0.36	119	0.10–0.24	0.09–0.11	0.24–0.25
		*E.m.	0.19	0.05	0.19	0.06	0.34	99	0.10–0.28	0.09–0.12	0.27–0.29
Calcium (mg/dL)	9–12	*L.a.	10.81	0.91	10.70	8.20	15.00	117	9.48–11.92	9.32–9.64	11.75–12.10
		*E.m.*	10.42	0.99	10.30	8.50	16.00	101	9.06–11.59	8.87–9.25	11.42–11.78
Carbon dioxide (mEq/L)	20–28	*L.a.*	20.25	3.12	20.10	13.60	31.20	121	14.50–25.57	13.84–15.11	24.87–26.30
		*E.m.*	20.57	3.17	20.35	15.40	41.00	102	15.47–24.90	14.83–16.11	24.23–25.66
Chloride (mEq/L)	100–115	*L.a.*	90.14	8.09	89.00	74.00	131.00	117	77.71–99.77	75.96–79.57	98.33–101.56
		*E.m.	94.10	11.98	91.00	74.00	169.00	102	72.38–107.34	69.38–75.28	104.38–110.22
Cholesterol (mg/dL)	26–68	*L.a.	62.45	13.25	61.00	23.00	99.00	117	39.89–83.35	37.09–42.36	80.50–86.34
		*E.m.*	40.63	12.71	40.00	25.00	144.00	103	24.49–54.73	22.45–26.51	52.70–56.80
Creatine kinase (IU/L)	50–250	*L.a.	185.57	224.61	146.00	48.00	2422.00	123	0.00–305.43	0.00–12.86	273.03–340.35
		*E.m.*	112.65	121.43	75.50	4.00	771.00	98	0.00–203.27	0.00–0.00	171.52–237.80
Creatinine (mg/dL)	1.0–2.0	*L.a.*	1.36	0.29	1.30	0.70	2.50	117	0.91–1.68	0.85–0.97	1.62–1.73
		*E.m.*	1.39	0.37	1.30	0.90	3.70	100	0.86–1.79	0.81–0.92	1.73–1.88
Gamma glutamyl transferase (U/L)	4–35	*L.a.	18.08	3.52	18.00	9.00	28.00	118	11.84–23.36	11.16–12.61	22.56–24.21
		*E.m.*	15.13	5.54	13.00	8.00	39.00	97	5.42–21.08	4.07–6.31	19.54–22.54
Glucose (mg/dL)	60–116	*L.a.*	79.98	13.16	80.00	49.00	137.00	119	58.07–99.87	55.19–60.84	97.32–102.36
		*E.m.*	78.49	18.39	76.00	37.00	137.00	96	47.88–104.88	44.04–51.55	100.02–109.72
Iron (μg/dL)	60–150	*L.a.	79.42	25.63	77.00	15.00	190.00	120	35.12–117.55	30.02–40.54	111.70–123.40
		*E.m.*	59.02	19.76	56.00	13.00	123.00	100	22.53–92.47	17.96–26.77	87.60–98.00
Lactate dehydrogenase (IU/L)	*250–500*	*L.a.	263.13	90.08	259.00	76.00	673.00	119	112.90–393.29	95.43–129.97	376.24–410.99
		*E.m.*	180.03	110.62	158.00	10.00	711.00	99	0.10–303.21	0.00–23.58	277.41–329.26
Magnesium (mg/dL)	*1.4–2.6*	*L.a.	2.18	0.31	2.10	1.40	3.30	120	1.61–2.69	1.54–1.66	2.63–2.77
		*E.m.*	1.96	0.26	1.90	1.50	3.10	101	1.45–2.35	1.37–1.48	2.24–2.41
Phosphorus (mg/dL)	*4.0–6.0*	*L.a.	4.67	0.77	4.60	2.60	8.00	121	3.34–5.92	3.18–3.50	5.77–6.08
		*E.m.*	4.46	0.85	4.40	2.10	7.10	96	3.05–5.55	2.86–3.26	5.36–5.74
Potassium (mEq/L)	*3.0–6.0*	*L.a.	4.92	0.48	4.90	3.80	6.40	118	4.09–5.67	4.00–4.19	5.57–5.78
		*E.m.*	4.81	0.80	4.60	3.70	9.50	99	3.63–5.63	3.48–3.77	5.47–5.80
Sodium (mEq/L)	*120–140*	*L.a.*	131.58	11.16	129.00	111.00	183.00	116	115.15–142.22	112.54–117.61	139.90–144.78
		*E.m.	136.30	16.46	131.00	107.00	235.00	100	106.98–153.73	102.89–111.00	149.95–157.78
Total protein (g/dL)	*6–12*	*L.a.*	8.19	0.83	8.20	6.30	12.90	118	6.98–9.35	6.82–7.15	9.19–9.50
		*E.m.	8.44	0.79	8.50	6.00	10.10	100	7.10–9.90	6.89–7.30	9.72–10.10
Triglycerides (mg/dL)	*15–60*	*L.a.*	34.20	21.85	28.00	5.00	140.00	119	0.00–60.05	0.00–0.30	54.61–65.35
		*E.m.	37.83	19.73	36.00	0.00	99.00	98	2.68–66.31	0.00–6.96	61.86–70.94
Urea nitrogen (mg/dL)	*5–20*	*L.a.*	6.43	2.67	7.00	1.00	14.00	120	1.42–11.16	0.87–1.92	10.62–11.72
		*E.m.	11.26	4.44	11.00	4.00	45.00	102	5.28–16.20	4.51–6.10	15.41–16.99

^a^ Fowler, M.E., Mikota, S.K., 2006. *Biology, Medicine, and Surgery of Elephants*, First Edition. Blackwell Publishing, Ames, Iowa, USA. ^b^ Number of samples used for value range calculation after outlier removal. * Species has higher concentrations at *p* < 0.05 level.
